# Costs and benefits of social connectivity in juvenile Greylag geese

**DOI:** 10.1038/s41598-019-49293-9

**Published:** 2019-09-06

**Authors:** Georgine Szipl, Marie Depenau, Kurt Kotrschal, Josef Hemetsberger, Didone Frigerio

**Affiliations:** 1 Core Facility Konrad Lorenz Forschungsstelle for Behaviour and Cognition, University of Vienna, Fischerau 11, 4645 Gruenau im Almtal, Austria; 2 Department of Behavioural Biology, University of Vienna, Althanstrasse 14, 1090 Vienna, Austria; 3 Department of Behavioural Biology, University of Osnabrueck, Barbarastrasse 11, 49076 Osnabrueck, Germany

**Keywords:** Reproductive biology, Animal behaviour

## Abstract

Living in groups has various advantages and disadvantages for group members. We investigated the fitness consequences of early social connectivity (normalized Freeman degrees based on nearest neighbour data), physiology (levels of excreted corticosterone metabolites assayed from droppings), and agonistic interactions in a group of free-ranging greylag geese (*Anser* *anser*). Forty-four greylag geese below 3 years of age were observed in three different seasonal phases: during the re-aggregation of the flock in autumn, at the end of the winter and during the forthcoming breeding season. We show that corticosterone metabolite levels and initiated and received aggression increased with increasing social connectivity. Individuals had higher connectivity scores in the winter flock than during the mating and breeding seasons. One-year old juveniles were more connected than 2- and 3-year old individuals. In addition, we examined the link between social connectivity during early development and reproductive success several years later. We found that birds with greater connectivity early in life attempted to breed at a younger age. Furthermore, successful breeders with higher early connectivity scores had higher numbers of fledged goslings. Our results show that social context in early life stages may have long-term effects on individual fitness.

## Introduction

The adaptive value of group living has long been documented, especially with emphasis on the costs and benefits involved^1^. The benefits range from increased vigilance and predator defense to better food accessibility, while the costs include increased competition over resources, and a higher risk of disease transmission. Another important cost in group-living vertebrates are social interactions, which were shown to be among the most potent stressors affecting behaviour, physiology, immune status and fertility of individuals^2–4^. At the same time, social partners are important stress buffering agents and can contribute to lowering costs of social life caused by stress^5^. The hypothalamus-pituitary-adrenal (HPA) axis modulates the hormonal response during stressful situations^6,7^, and social context has been shown to play a major role in eliciting hormone-dependent behaviours (reviewed in^8^). Depending on the context, social challenges may affect individuals differently^2,4,9^. Social isolation, for example, might be a powerful stressor in group-living species. In mammals, social isolation can lead to an increase in cortisol levels, as shown for instance in immature common marmosets (*Callithrix jacchus*)^10^, and in group-living juvenile male African striped mice (*Rhabdomys pumilio*)^11^. Also in birds, isolation from the social group resulted in higher corticosterone levels in gregarious European starlings (*Sturnus vulgaris*)^12^ and zebra finches (*Taeniopygia guttata*)^13^. As shown in several bird species, the complex interplay between social behaviour and corticosterone may be modulated by sex^14^, body size^15^, daily cycles^16^, environmental factors^17^, but also by social integration^18^, and developmental history^19^. For example, ravens (*Corvus corax*) that were socially well embedded (i.e. had more allies) were shown to excrete higher levels of corticosterone metabolites when separated from the social group, while the opposite was found for poorly integrated individuals, that “relaxed” when isolated from conspecifics^18^. Zebra finches exposed to corticosterone early in life were shown to have weaker associations to their parents, but instead higher numbers of association with other flock mates later in life^19^. Both studies highlight that the structure of social groups, as well as the position of an individual within the social group, which was assessed using social network analysis (SNA) can have important fitness-relevant consequences. SNA was shown to be a useful tool to investigate the costs and benefits of sociality on individual fitness^20^.

Social associations can aid in maximizing one’s inclusive fitness, and although the underlying evolutionary mechanism is still unclear (e.g. kin selection^21^, reciprocal altruism^22^, mutualism), measures of fitness are often based on the number of offspring and close relatives. The influence of social bonds on reproductive success has mostly been investigated in primates. In vervet monkeys (*Chlorocebus pygerythrus*), young adult females had stronger social bonds to their mother than to other group members, and females with mothers produced more surviving offspring, while females without these bonds were more often the target of aggression^23^. Similarly, in baboons (*Papio cynocephalus ursinus*), females with stronger bonds to their mothers and adult daughters had higher offspring survival^24^. Also in males, social bonds may lead to high reproductive success due to coalition formation and increased rank, as shown in Assamese macaques (*Macaca assamensis*^25^). Most of these studies highlight a complex interplay between social bonds, dominance rank, and reproductive success. Outside the primate order, a link between fitness benefits and social bonds was shown for example in feral horse (*Equus ferus*) mares, where social integration was positively linked to foal birth rates^26^. In bottlenose dolphins (*Tursiops* sp.), calving success of females was positively linked to social and genetic factors^27^, indicating that sociality influences fitness components.

Greylag geese form large flocks with complex social relationships^28^. Families dominate pairs in aggressive encounters and pairs tend to win against single individuals^29–31^. Such social bonds modulate coping with stressful situations via active and passive (i.e. “emotional”) social support^32^. Social support (i.e. the stress reducing effect of the presence of a social ally^33^), during agonistic encounters may indeed be considered as a main factor influencing the maintenance of long lasting bonds between offspring and their parents^34^. In fact, the young of the year stay with their parents for an entire year, and eventually join them again if they fail to reproduce^35^. Furthermore, females and juveniles seem to benefit from the effects of social support, probably because they are lower in rank than their partners and males in general: females and juveniles were shown to win more agonistic encounters, and juveniles fed longer and were less often attacked when they received social support^34,36,37^. Greylag geese exhibit biparental care, and parenting males were shown to excrete higher levels of corticosterone metabolites than singletons^38^. During their development from fledging at the age of approx. 8 to 10 weeks to sexual maturity between 2 and 3 years of age^31^, juveniles are challenged to acquire and maintain a stable social position within the flock, as this will allow them to gain more weight, and thus better access to high quality resources^39^. A high rank was also shown to increase reproductive success in bar-headed geese (*Anser indicus*), likely because of the high dominance in parental males, which enabled them to acquire and defend a breeding site^40^.

In the present study, we investigated the interplay between early social connectivity, calculated as centrality based on the number of nearest neighbours in a SNA,  and physiology, expressed as levels of excreted corticosterone metabolites (CM), in 1-year old juveniles and immature (2- and 3-year old) greylag geese in a non-migratory and free-roaming flock in Upper Austria. Three different biologically relevant phases of the flock life were investigated: during the re-aggregation of the flock in autumn and winter (November-December), at the beginning of the mating season at the end of the winter (January-February) and during the forthcoming breeding season (March-April). Behavioural observations on 44 goslings hatched between 2013 and 2015 were conducted from November 2015 to April 2016 using the software Prim8 Mobile (mobile computing to record nature, http://www.prim8software.com)^41^. Initiated and received agonistic behaviours were recorded, and social connectivity was assessed using nearest neighbour data (spatial proximity between flock members). Furthermore, we tested whether social connectivity early in life would predict future reproductive success, which we measured as the number of fledged goslings per year. We expected increased levels of CM with increasing connectivity, as the acquisition of a social position in the flock may involve stressful and energetically costly agonistic interactions. Furthermore, as geese usually loosen their social bonds with their parents at the end of their first year^31^, a gradual decrease in social connectivity was expected with age. Likewise, social connectivity was expected to be higher during stable winter flocking as compared to the breeding season when family-bonds break up and pair bonds may be formed^31^. With regard to future reproductive success, we expected to find higher numbers of fledged goslings from individuals that showed high early social connectivity, probably due to access to high quality nesting and foraging sites.

## Results

**Figure 1:**
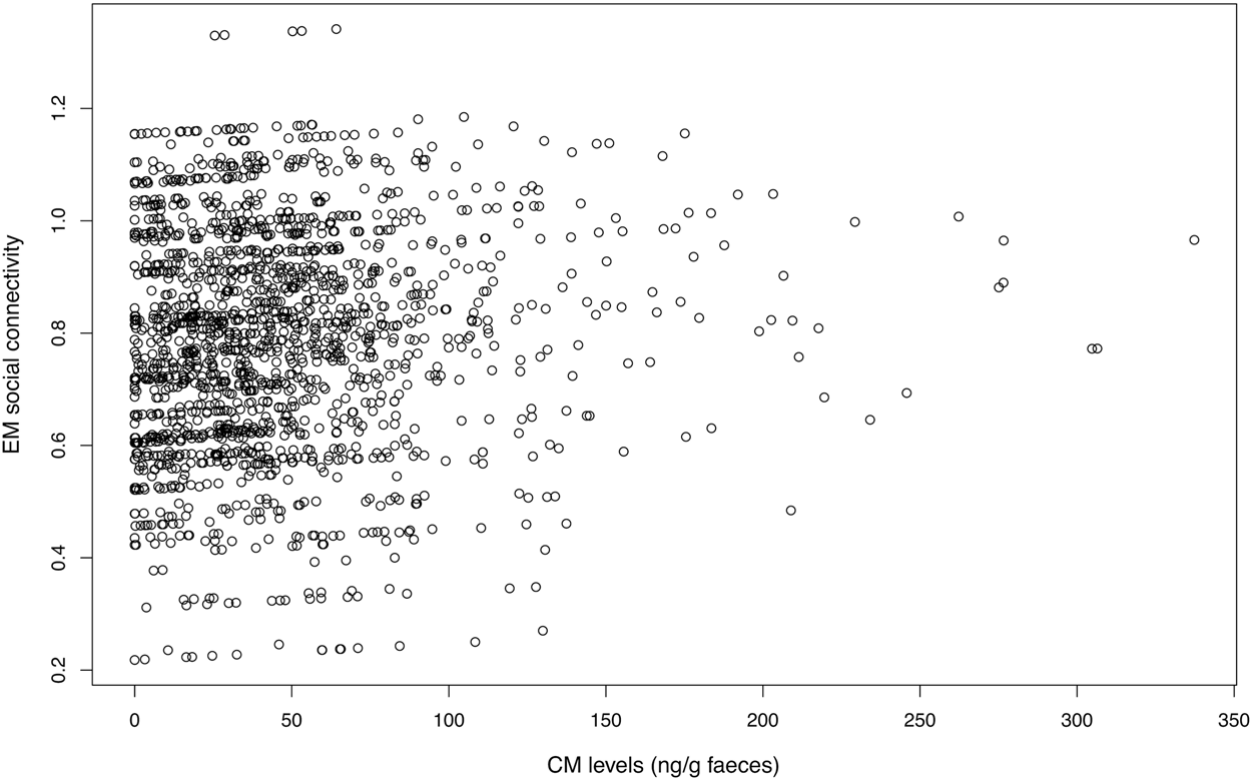
Scatter plot showing estimated mean (EM) social connectivity and CM levels (ng/g faeces).

**Figure 2:**
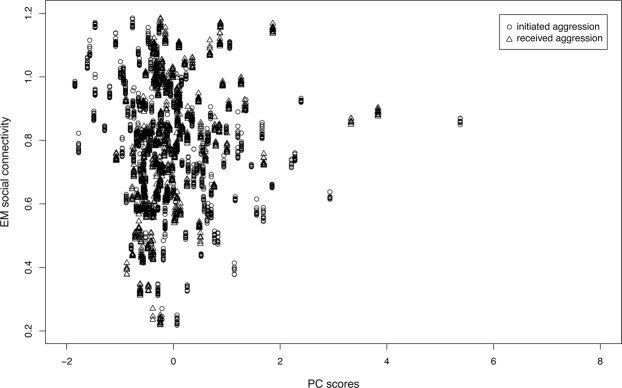
Estimated mean (EM) social connectivity and Principal Component (PC) scores of initiated (squares) and received (triangles) aggression.

**Figure 3:**
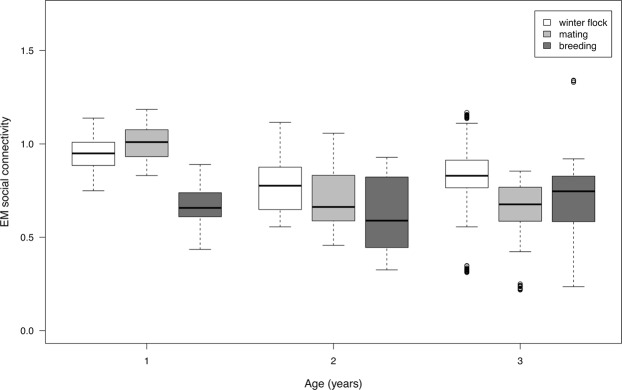
Estimated mean (EM) social connectivity and gosling age in years, separately for the three seasons investigated.

Social connectivity was significantly linked to CM levels, initiated and received aggression, season, age, and the season by age interaction (Table 1). CM levels (Fig. 1) as well as initiated and received aggression (Fig. 2) increased with increasing social connectivity. Juvenile individuals had higher connectivity than 2 and 3-year olds, and social connectivity was higher in the winter flock as compared to the mating period and the breeding season. The interaction between season and age showed a high connectivity during the winter and the mating season in juveniles, while social connectivity gradually decreased in 2- and 3-year old individuals from winter throughout the mating and breeding season (Fig. 3).

**Table 1:** Estimated means (EM), standard errors (SE), t values and confidence intervals (CI) for all coefficients in the LMM.

	EM	SE	t value	CI (2.5%)	CI (97.5%)
(Intercept)	0.6882	0.1036	6.64	0.7480	1.1520
CM (ng/g)	0.0003	0.0001	2.35	0.0001	0.0005
Received aggression	0.0460	0.0077	6.00	0.0304	0.0607
Initiated aggression	0.0873	0.0079	11.08	0.0720	0.1028
Season (mating)^a^	0.1877	0.0292	6.44	0.1307	0.2449
Season (breeding)^a^	−0.4460	0.0373	−11.94	−0.5179	−0.3713
Age	−0.0827	0.0334	−2.48	−0.1486	−0.0082
Sex^b^	−0.0045	0.0485	−0.09	−0.0966	0.0950
No. of siblings	0.0234	0.0213	1.10	−0.0174	0.0643
Season(mating):Age	−0.1098	0.0133	−8.25	−0.1359	−0.0839
Season(breeding):Age	0.1145	0.0170	6.74	0.0807	0.1473

Set as reference points:

^a^Season (winter flock).

^b^Females

“:” denote interactions.

Post-hoc Mann-Whitney U tests (Table 2) showed that in juveniles, social connectivity was significantly higher during winter and the mating season as compared to the beginning of the breeding season (winter flock vs. breeding season: U = 208.0, p < 0.0002; mating vs. breeding season: U = 210.0, p < 0.0002). In 3-year olds, social connectivity was significantly higher in winter as compared to the mating season (U = 153.0, p = 0.0318).

**Table 2:** Post-hoc Mann-Whitney U tests of seasonal differences in estimated mean social connectivity in 1-, 2- and 3-year old greylag geese (‘*N*_*1*,*2*_’ indicate sample sizes per season per age class). Original *P* values and values adjusted after Benjamini & Hochberg (*P*_*FDR*_) are shown. Significant differences after controlling for false discovery rate are indicated in bold.

Pairwise comparisons	*N*_*1,2*_	*U*	*P*	*P*_*FDR*_
***Juveniles***				
winter flock - mating season	15,15	73.0	0.1064	0.1064
**winter flock - breeding season**	**15,14**	**208.0**	**<0.0001**	**<0.0002**
**mating - breeding season**	**15,14**	**210.0**	**<0.0001**	**<0.0002**
***2-year olds***				
winter flock - mating season	15,15	139.0	0.2854	0.2854
winter flock - breeding season	15,14	154.0	0.0328	0.0984
mating - breeding season	15,14	140.0	0.1337	0.2006
***3-year olds***				
**winter flock - mating season**	**14,14**	**153.0**	**0.0106**	**0.0318**
winter flock - breeding season	14,14	133.0	0.1139	0.1708
mating - breeding season	14,14	79.0	0.4013	0.4013

**Figure 4:**
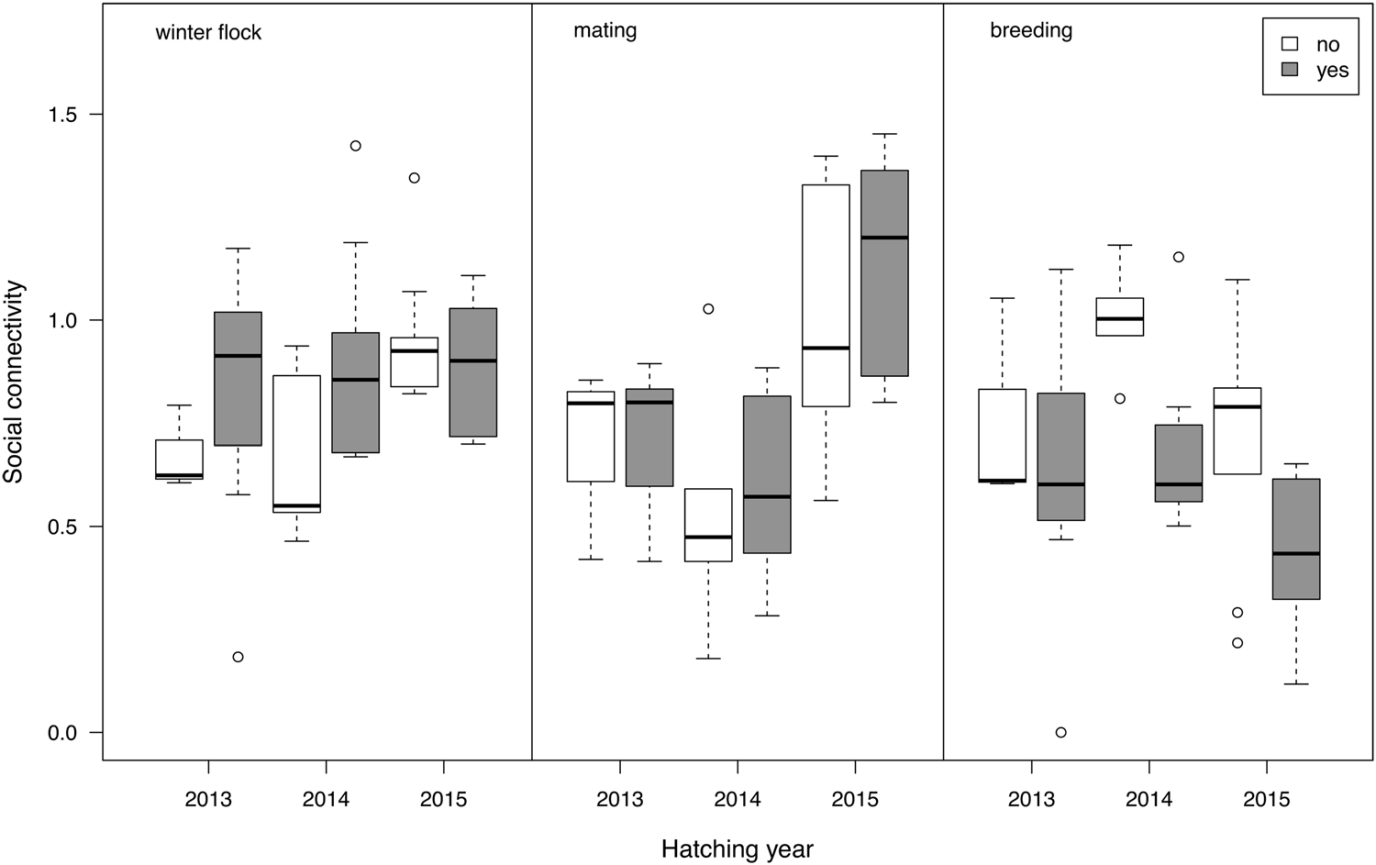
Social connectivity scores and breeding attempts (no = white boxes, yes = grey boxes) for goslings hatched in 2013 (3-year old), 2014 (2-year old), and 2015 (juveniles), separately for the three seasons (winter flock, mating season, breeding season) in focus.

When examining how early social connectivity was linked to future reproduction, we found that the interaction between social connectivity and season significantly affected the likelihood to attempt breeding in 2-year old geese (Table 3). Individuals that made a breeding attempt up until 2018 showed a non-significant trend towards having greater social connectivity in the winter flock of 2015, but showed significantly lower connectivity in the breeding season in 2016 (cp. Table 4 and Fig. 4). These findings indicate that individuals that attempted to breed up until 2018 showed a high connectivity during the winter flock season in 2015, while individuals that were still within a group and highly connected during the breeding season in 2016 did not attempt to breed in the consecutive years.

**Table 3:** Coefficients of the logistic regression investigating breeding attempts (yes/no), with estimated means (EM), standard errors (SE), z values and significance levels (p), separately for juveniles, and 2- and 3-year olds. Significant coefficients are indicated in bold.

	EM	SE	z value	p
***Juveniles***				
(Intercept)	2.036	3.455	0.59	0.556
Social connectivity	−2.653	3.744	−0.71	0.479
Season (mating)^a^	−4.286	4.112	−1.04	0.297
Season (breeding)^a^	−0.312	3.766	−0.08	0.934
Social connectivity:Season(mating)	4.356	4.230	1.03	0.303
Social connectivity:Season(breeding)	−1.423	4.514	−0.32	0.753
***2-year olds***				
(Intercept)	−3.494	2.690	−1.30	0.194
Social connectivity	5.462	3.603	1.52	0.130
Season (mating)^a^	3.479	3.075	1.13	0.258
**Season (breeding)** ^**a**^	**11.241**	**4.616**	**2.44**	**0.015**
Social connectivity:Season(mating)	−4.216	4.375	−0.96	0.335
**Social connectivity:Season(breeding)**	**−13.932**	**5.542**	**−2.51**	**0.012**
***3-year olds***				
(Intercept)	−0.469	1.996	−0.24	0.814
Social connectivity	2.341	2.607	0.90	0.369
Season (mating)^a^	1.231	3.262	0.38	0.706
Season (breeding)^a^	2.808	2.675	1.05	0.294
Social connectivity:Season(mating)	−1.576	4.433	−0.36	0.722
Social connectivity:Season(breeding)	−3.854	3.454	−1.12	0.265

Set as reference point:

^a^Season (winter flock).

“:” denote interactions.

**Table 4:** Coefficients of the logistic regression investigating breeding attempts (yes/no) in 2-year old geese, with estimated means (EM), standard errors (SE), z values and significance levels (p), separately for the three seasons. Significant results are indicated in bold.

	EM	SE	z value	p
***Winter flock***				
(Intercept)	−3.494	2.690	−1.30	0.194
Social connectivity	6.962	3.603	1.92	0.053
***Mating season***				
(Intercept)	−0.015	1.490	−0.01	0.992
Social connectivity	1.246	2.482	0.50	0.616
***Breeding season***				
(Intercept)	7.746	3.751	2.07	0.039
**Social connectivity**	**−8.470**	**4.211**	**−2.01**	**0.044**

Early social connectivity did not affect the likelihood to breed successfully (Table S1 in the Supplementary Information). However, within the successful breeders, we found a significant positive effect of social connectivity in the winter flock in 2015 on the number of fledged goslings: individuals with higher connectivity in the winter flock fledged more goslings than individuals with lower connectivity (Table 5). No effect of social connectivity on the number of fledged goslings could be found for the mating or the breeding season 2016 (cp. Table 5).

**Table 5:** Coefficients of the regression investigating the number of fledged goslings, with estimated means (EM), standard errors (SE), t values and significance levels (p), separately for the three seasons. Significant results are indicated in bold.

	EM	SE	t value	p
***Winter flock***				
(Intercept)	−0.888	0.765	−1.16	0.279
**Social connectivity**	**2.128**	**0.848**	**2.51**	**0.036**
***Mating season***				
(Intercept)	0.139	0.762	0.18	0.860
Social connectivity	1.115	0.983	1.13	0.290
***Breeding season***				
(Intercept)	1.220	0.667	1.83	0.105
Social connectivity	−0.449	0.919	−0.49	0.638

## Discussion

Social connectivity during early developmental stages was affected by CM levels, agonistic interactions, the age of the individuals, and seasonal effects. Excreted CM levels as well as initiated and received agonistic interactions increased with increasing social connectivity. Highest scores of connectivity were found during winter, and 1-year old individuals were better connected than 2- and 3-year olds. Most importantly, early social connectivity was an indicator of future breeding attempts and the number of goslings fledged up until 2018, and thus indicates that high social connectivity early in life may provide important fitness benefits.

CM concentrations and initiated and received aggression increased with increasing social connectivity, indicating that having more social connections also means having more agonistic interactions, potentially causing increased CM. Although dominance was not measured in the present study, one possible explanation could be that the observed pattern reflects the consequences of a dominance hierarchy that is established in juveniles and 2- and 3-year old geese during flocking in winter. This is further supported by our finding that social connectivity of our focal individuals was generally higher in the winter flock than in the mating and breeding seasons. In many group-living vertebrates, individuals which engage in agonistic interactions to gain and maintain a high rank show higher CM concentrations than subordinate ones, especially in groups with instable hierarchies (reviewed in^42^). Greylag goose flocks split up in pairs for breeding, and depending on their breeding success, re-join the flock together with their goslings as families, or as pairs without goslings^31^. In general, families outrank pairs and singletons^29–31^, but a dominance hierarchy has to be established between the different families every year. This seasonal flocking with varying social status may result in an unstable hierarchy, where families have to re-establish their rank constantly, which may lead to more agonistic interactions in high-ranking families, and thus higher CM levels in family members. However, the relationship between CM levels and dominance rank is complex, and depends amongst others on the social system^43^. As social connectivity was highest in juveniles, and agonistic interactions and CM levels declined with declining social connectivity, our findings may also imply that an individual’s position in the flock is established as a juvenile. As 2- and 3-year olds had lower social connectivity as compared to juveniles, it is likely that the first establishment of flock position has long-lasting effects, and the re-establishment in the next years is based on the initial flock position. Yet, members of larger families can support each other better in conflicts and may be better able to maintain their high-ranking position within the flock. In barnacle geese, for example, larger broods were shown to be more dominant^44^, and to have better access to resources^45^. In Greylag geese, it was further shown that individuals that received passive social support by an ally (human foster parent) won a higher percentage of agonistic interactions, but at the same time showed higher CM levels^46^. CM concentrations are known to vary also with several non-social factors. The type and quality of available food as well as the size of the digestive tract affect gut passage time and excrement mass (reviewed in^47^). However, the goose population in this study is non-migratory, and supplemented with the same type of food twice a day. In addition, data collection took place during autumn/winter and spring, when little to no additional food is available. Thus, we can exclude variation in CM concentrations due to differences in diet (e.g. some geese feeding more on grass and less on supplemented pellets and grain).

One-year old individuals in our study were, in general, more connected than 2- and 3-year olds. This finding highlights the importance of the family unit for the integration of the goslings into the flock. One-year old goslings join the flock together with their parents, and stay with their parents or siblings throughout the winter^28^. Juveniles may aggregate in sibling groups during the mating season, and may even continue to stay with them and/or their parents for a second year^31^. However, the number of siblings did not have a strong effect on early social connectivity (cp. Table 1), indicating that nearest neighbours were not solely kin, but also other members of the flock. This suggests that 1-year old individuals associated not only with their siblings, but probably join other non-breeding individuals during the mating season. This may well be the reason for the high connectivity of juveniles also during the mating season as compared to 2- and 3-year old goslings, which showed low connectivity during the mating and the breeding season, most likely because they were mated with another goose and formed a pair bond, and thus had fewer nearest neighbours. The integration of juveniles into the social group was shown to depend on close kin also in other species: in juvenile Spectacled Parrotlets (*Forpus conspicillatus*), strong bonds with kin were shown to be vital, as juveniles without social support from siblings were lower in rank^48^. In moose (*Alces alces shirasi*) calves, integration into the loose winter aggregations was shown to depend on the mother, and juveniles without their mother were not integrated, and as a consequence, did not survive the winter^49^.

When investigating if early social connectivity would predict future breeding performance, we found that 2-year old individuals which showed high levels of social connectivity during the winter flock in 2015 were more likely to attempt breeding up until 2018 than those with low connectivity. As the lifetime number of breeding events increases with an early onset of reproduction^50^, birds that start reproduction early in life will have a higher change to increase their lifetime reproductive success, and hence their inclusive fitness^21^, although some studies have shown that early reproduction comes at the cost of faster senescence^51,52^. Within the birds that had attempted and succeeded breeding up until 2018, the number of goslings fledged was positively linked to early social connectivity in the winter flock in 2015, indicating that early social connectivity can provide benefits for future breeding.

Our results show that also in highly social birds, the social context in early stages of life has profound effects on future reproductive success. Young individuals joining a social group may benefit from social support of family members to acquire a position within the group that will ultimately enable them to start reproduction early in life, and thus provide important fitness benefits.

## Methods

### Study area and animals

A free-ranging flock of greylag geese with marked individuals and known life-histories was investigated. The original non-migratory flock was introduced into the Upper Austrian valley of the River Alm in 1973 by Konrad Lorenz and co-workers^31,53^. Individuals are unrestrained and roam the valley between the Lake Alm in the south, where they roost at night, and the Research Station (KLF), where they are supplemented with pellets and grain twice a day on the meadows around the house. At the time of data collection, the flock consisted of approx. 190 greylag geese, which were all individually marked using different combinations of coloured leg bands. Individual life history data and social backgrounds of all individuals have been continuously monitored since 1973. Eggs of foreign flocks are regularly taken for hand-raising purposes, and hand-raised geese are successfully introduced into the flock^54^, thereby assuring that genetic variability in the flock is maintained. The birds are habituated to the close presence of humans, which allows collection of behavioural and physiological data without influencing levels of excreted immunoreactive corticosterone metabolites^55^.

### Data collection

Data were collected from November 2015 to April 2016, and the data collection was split into three seasons^56^. Season one included November and December 2015 and was considered the time when the geese build a stable flock during winter. Season two lasted from January to February 2016 and covered the mating season prior to breeding and the loosening of the parent-offspring bonds. Season three comprised the months March and April 2016, and included the onset of the breeding season, where the flock dissolves and the breeding pairs remain by themselves. Throughout all three seasons, both behavioural protocols and individual droppings were collected of all focal individuals.

Focal birds consisted of 44 individuals out of 78 hatched goslings between 2013 and 2015 (see Table S2 in the Supplementary Information). They were grouped in annual cohorts of 14 birds (8 males, 6 females) from 2013, and 15 birds (8 males,7 females) from 2014 and 2015, respectively. Two focal individuals disappeared during data collection in the third season, resulting in 42 focal individuals (14 birds per annual cohort). Focal birds belonged to 15 different families within the flock. At the beginning of data collection, the focal individuals within the flock were either single individuals, i.e. juveniles that were neither joining kin or non-kin conspecifics, nor had a partner (N = 9; 6 males, 3 females), individuals joining the parents in a primary (N = 15; 8 males, 7 females) or secondary family (N = 4; 1 male, 3 females) and paired individuals (N = 16; 9 males, 7 females).

Focal individuals were observed continuously for 10 minutes after the feeding sessions (i.e. after the food was depleted by the geese) in the morning (8 am) or in the afternoon (3 pm). Behavioural observations were conducted using the software Prim8 Mobile (mobile computing to record nature, http://www.prim8software.com/)^41^. The behaviours in focus were initiated and received agonistic social interactions of varying intensity, and nearest neighbour data (spatial proximity between flock members). A mean number of 17.41 ± 2.05 (SD) protocols were taken per focal individual (see Table S3 in the Supplementary Information).

Droppings were collected from 10 am to 12.30 pm after feeding in the morning to avoid the early morning peaks of adrenal activity^16,57^ and to obtain an estimate of the daytime baseline levels. In geese, droppings represent an integrated, proportional record of the plasma level within a time period of 2–4 hours prior to defecation^58^. Samples were frozen within an hour after collection and stored at −20 °C^38,59^. As geese defecate approximately every 20 minutes^58^ and variation between individual samples tends to be high, 1699 droppings were analysed in total. The number of dropping samples per individual ranged from 29 to 47 (mean ± SD: 38.61 ± 4.22 droppings per individual; for details see Table S3 provided in the Supplementary Information).

### Analysis of droppings

In order to obtain information about CM levels, droppings were analysed using an enzyme immunoassay according to a method developed and validated for greylag geese^60^. Details about the procedure and cross-reactivity are already published^17,38^. The mean intra- and inter-assay coefficients of variance for the faecal samples amounted to 5.48% (<15%) and 6.58% (25%), respectively.

### Statistical analyses

A Principal Component Analysis (PCA) was conducted using the package GPA rotation (version 2014.11-1^61^) in R (version 3.5.0^62^). The frequencies of agonistic behaviours towards other geese were corrected for the number of observations by dividing the frequencies by the number of observations per focal individual.

The corrected frequencies of active (‘attack’ and ‘threat’) and passive (‘avoid’ and ‘escape’) agonistic behaviours per individual per season were used to run a PCA with a varimax rotation. The four agonistic behaviours loaded on two principal components (PC), and explained 63% of the variance in total (Table 6). The passive agonistic behaviours ‘avoid’ and ‘escape’ had high positive loadings on PC 1. In addition, ‘threat’ loaded with a moderate but negative loading on PC 1. Thus, this component was termed ‘*received aggression component*’. PC 2 contained only the active agonistic behaviour ‘attack’, and was termed ‘*initiated aggression component*’. Component scores were extracted and used in the models.

**Table 6:** Component matrix of the PCA for the frequencies of active and passive agonistic behaviour. Highest factor loadings on one component are indicated in bold.

Behaviour	Principal Components	
	PC1	PC2
avoid	**0.80**	−0.04
escape	**0.76**	0.36
threat	**−0.47**	0.26
attack	0.0	**0.94**
% variance explained	36	27

Nearest neighbour data was summarized for each season and extracted as a matrix including the frequencies of dyadic nearest neighbour incidences. For each season, weighted, undirected social networks were constructed, and normalized Freeman degrees were extracted for each focal individual based on the nearest neighbour data from focal observations. Normalized Freeman degrees were calculated using the software UCINET 6 for Windows^63^. The Freeman degree describes the number of nodes a given node is adjacent, i.e. directly connected to^64^. Thus, the degree is a measure of gregariousness or popularity, and will be used as a measure of social connectivity. The normalized degree was the degree divided by the maximum degree possible.

A linear mixed-effect model (LMM) was used to investigate the effects of CM, age and season on social connectivity. The LMM was calculated using the lme4 package (version 1.1–17^65^) in R (version 3.5.0^62^). The normalized Freeman degree was used as response variable. CM values (ng/g faeces), the geese’s age (in years) and sex, season, the scores of initiated and received aggression components, and the number of siblings from that year were entered as fixed effects. In addition, a two-way interaction between age and season was entered in the model. Collinearity between fixed factors was tested and ruled out prior to model fitting by calculating Variance Inflation Factors^66^. The identity of the focal individual and the mother nested within the years were used as random effects to account for repeated measures and siblings (i.e. goslings having the same mother) from several consecutive years. The full model explained variation in the data significantly better than the Null model (Likelihood Ratio Test: γ^2^ = 557.3, df = 10, p < 0.0001). Post-hoc pairwise comparisons were calculated using Mann-Whitney U tests on sample sizes corrected for repeated measures. To control for the false discover rate (FDR), Benjamini & Hochberg adjustment of all p values was applied^67^.

To investigate the relationship between early social connectivity and future reproductive success, binomial categories (yes/no) were created for breeding attempts and breeding success up until the breeding season 2018. Reproduction was measured as the number of fledged goslings divided by age (in years) to correct for the varying number of opportunities to reproduce. Differences in early social connectivity between those individuals that had attempted breeding and those that had not and successful and unsuccessful breeders were tested separately for individuals hatched in 2013, 2014 and 2015 due to collinearity between age and social connectivity. The binomial response variables breeding attempt (yes/no) and breeding success (yes/no) were analysed using logistic regression models. Social connectivity, season, and their two-way interaction were entered as fixed effects. If the interaction between social connectivity and season showed a significant effect, data was split for the three different seasons, and separate regressions were conducted using social connectivity as a fixed effect.

Within those individuals that had attempted to breed up until 2018 (n = 27), we further differentiated between individuals that had bred successfully (n = 10) and those that had not (n = 17). Successful breeding was defined as having fledged offspring successfully. Linear regressions were calculated with the number of goslings fledged up until 2018 (corrected for the age of the parent) as a response variable, and social connectivity, season, and their two-way interaction as fixed effects. To further investigate the interaction between season and social connectivity, the data set was split for the three seasons and separate regressions were conducted.

### Animal welfare note

The permission to keep greylag geese for scientific purposes according to §16 of the Austrian Animal Experiments Act (Federal Law Gazette No. 114/2012) was issued by the Austrian Federal Ministry of Science, Research and Economy (BMWFW) under the license number BMWFW-66.006/0011-WF/II/3b/2014. No additional approvals were required because the study was non-invasive, and did not fall under the Austrian Animal Experiments Act §2. This research adheres to the ASAB/ABS Guidelines for the Use of Animals in Research^68^.

## Supplementary information

Supplementary Information

## Data Availability

The datasets generated and analysed during the current study are available from the corresponding author on request.
