# Supplementary material for: Costs and benefits of social connectivity in juvenile Greylag geese
Source: Sci Rep. 2019 Sep 6;9:12839. doi: 10.1038/s41598-019-49293-9 (PMC6731237; doi:10.1038/s41598-019-49293-9)
Supplement: Supplementary file 1 — Supplementary Information [file 41598_2019_49293_MOESM1_ESM.pdf]

Supplementary Information as referred to in

Costs and benefits of social connectivity in juvenile Greylag geese

Georgine Szipl, Marie Depenau, Kurt Kotrschal, Josef Hemetsberger, Didone Frigerio

Table S1. Coefficients of the linear regression investigating breeding success (yes/no), with estimated means (EM), standard errors (SE), z values and significance levels (p), separately for juveniles, and 2- and 3-year olds.

	EM	SE	z value	p
**Juveniles**				
(Intercept)	-10.244	10.015	-1.02	0.306
Social connectivity	10.359	10.283	1.01	0.314
Season (mating)[a]	9.731	10.798	0.90	0.367
Season (breeding)[a]	11.006	10.265	1.07	0.284
Social connectivity:Season(mating)	-10.266	10.831	-0.95	0.343
Social connectivity:Season(breeding)	-12.143	11.297	-1.08	0.282
**2-year olds**				
(Intercept)	0.432	2.575	0.17	0.867
Social connectivity	-0.942	2.825	-0.33	0.739
Season (mating)[a]	-0.397	3.315	-0.12	0.905
Season (breeding)[a]	-2.961	3.834	-0.77	0.440
Social connectivity:Season(mating)	0.205	4.373	0.05	0.963
Social connectivity:Season(breeding)	4.294	4.937	0.87	0.384
**3-year olds**				
(Intercept)	0.593	2.018	0.29	0.769
Social connectivity	-1.402	2.351	-0.60	0.551
Season (mating)[a]	2.183	3.388	0.64	0.519
Season (breeding)[a]	-1.371	2.416	-0.57	0.571
Social connectivity:Season(mating)	-3.364	4.456	-0.76	0.450
Social connectivity:Season(breeding)	1.749	2.989	0.59	0.558

Set as reference point:
[a]: Season (winter flock)
"：" denote interactions

Table S2. List of the 44 focal individuals including year of hatching, sex, information on the relatedness (i.e. the mother), and their pair partner.

**Name**	**Year of hatching**	**Sex**	**Mother**	**Partner**
Kaikos	2013	female	Kleine Hexe	JayLo (2013)
Kiribati	2013	male	Kleine Hexe	Iris (2014)
Bonsai	2013	female	Baggins	
Lacrima	2013	female	Leviathan	Brooklyn (2012)
Lenka	2013	female	Leviathan	Sinclair (2008)
Levuka	2013	male	Lando	
Dädalus	2013	male	Duftspur	Langschweif (2014)
Dardanos	2013	female	Duftspur	Jager (2012)
Taras	2013	male	Timber	Ibis (2014)
Timo	2013	male	Timber	Icecream (2014)
Thalia	2013	female	Timber	Lamborghini (2012)
Joe	2013	male	Judith	Bettina (2014)
JayLo	2013	male	Judith	Kaikos (2013)
Inti	2013	male	Ingeborg	Jamila (2014)
Kokosnuss	2014	male	Kleine Hexe	
Lennox	2014	male	Lando	
Lona	2014	female	Leviathan	
Trevor	2014	female	Timber	
Jurek	2014	male	Judith	
Jessica	2014	female	Judith	Bloody Mary (2012)
Icecream	2014	female	Ingeborg	Timo (2013)
Ingwer	2014	male	Ingeborg	
Langschweif	2014	female	Löwenherz	Dädalus (2013)
Lawrence	2014	male	Löwenherz	
Berta	2014	female	Barbados	
Bosse	2014	male	Barbados	Hermine (2014)
Halleluja	2014	male	Longshanks	
Hermine	2014	female	Longshanks	Bosse (2014)
John	2014	male	Jewel	
Kolumbien	2015	female	Kleine Hexe	
Kirgistan	2015	male	Kleine Hexe	
Lavender	2015	female	Leviathan	
Lederblümchen	2015	male	Leviathan	
Locke´s	2015	female	Löwenherz	
Longmorn	2015	male	Löwenherz	
Ismail	2015	female	Ingeborg	
Banane	2015	female	Barbados	
Blaubeere	2015	male	Barbados	
Hedwig	2015	male	Longshanks	
Jongsong	2015	female	Jewel	

GlenGrant	2015	male	Ginny	
Diamante	2015	female	Duftspur	
Demant	2015	male	Duftspur	
Benromach	2015	male	Baggins	

Table S3. Summary of focal observations and collected droppings per focal individual per season.

Focal individual	Number of focal observations						Number of droppings					
	Season 1	Season 2	Season 3	Sum	Mean	SD	Season 1	Season 2	Season 3	Sum	Mean	SD
Banane	8	5	4	17	5.67	1.70	16	15	15	46	15.33	0.47
Benromach	8	3	6	17	5.67	2.05	18	14	13	45	15.00	2.16
Berta	9	4	6	19	6.33	2.05	16	15	9	40	13.33	3.09
Blaubeere	8	5	2	15	5.00	2.45	19	14	14	47	15.67	2.36
Bonsai	8	5	7	20	6.67	1.25	16	13	10	39	13.00	2.45
Bosse	9	5	4	18	6.00	2.16	18	12	10	40	13.33	3.40
Daedalus	9	4	7	20	6.67	2.05	16	12	6	34	11.33	4.11
Dardanos	8	5	1	14	4.67	2.87	20	18	5	43	14.33	6.65
Demant	8	6	6	20	6.67	0.94	15	14	7	36	12.00	3.56
Diamante	8	5	NA	13	6.50	1.50	17	12	NA	29	14.50	2.50
GlenGrant	8	4	5	17	5.67	1.70	19	12	11	42	14.00	3.56
Halleluja	9	5	5	19	6.33	1.89	15	12	9	36	12.00	2.45
Hedwig	8	4	3	15	5.00	2.16	16	14	10	40	13.33	2.49
Hermine	9	5	4	18	6.00	2.16	15	11	9	35	11.67	2.49
Icecream	9	4	2	15	5.00	2.94	14	14	11	39	13.00	1.41
Ingwer	9	5	8	22	7.33	1.70	18	13	8	39	13.00	4.08
Inti	8	5	4	17	5.67	1.70	19	14	5	38	12.67	5.79
Ismail	8	5	4	17	5.67	1.70	16	12	11	39	13.00	2.16
JayLo	10	4	5	19	6.33	2.62	17	12	8	37	12.33	3.68
Jessica	9	5	8	22	7.33	1.70	18	14	15	47	15.67	1.70
Joe	8	4	5	17	5.67	1.70	16	15	9	40	13.33	3.09
John	9	5	NA	14	7.00	2.00	17	16	NA	33	16.50	0.50
Jongsong	8	5	4	17	5.67	1.70	16	14	8	38	12.67	3.40
Jurek	9	5	7	21	7.00	1.63	16	14	9	39	13.00	2.94

Kaikos	10	4	5	19	6.33	2.62	16	14	8	38	12.67	3.40
Kirgistan	8	4	3	15	5.00	2.16	16	14	11	41	13.67	2.05
Kiribati	8	4	5	17	5.67	1.70	16	14	12	42	14.00	1.63
Kokosnuss	9	6	5	20	6.67	1.70	17	12	11	40	13.33	2.62
Kolumbien	8	4	3	15	5.00	2.16	16	14	13	43	14.33	1.25
Lacrima	9	4	6	19	6.33	2.05	21	14	9	44	14.67	4.92
Langschweif	9	5	4	18	6.00	2.16	16	14	3	33	11.00	5.72
Lavender	8	5	4	17	5.67	1.70	15	13	8	36	12.00	2.94
Lawrence	9	5	4	18	6.00	2.16	16	14	5	35	11.67	4.78
Leberbluemchen	8	4	3	15	5.00	2.16	15	11	13	39	13.00	1.63
Lenka	8	5	4	17	5.67	1.70	15	13	3	31	10.33	5.25
Lennox	9	5	4	18	6.00	2.16	14	12	8	34	11.33	2.49
Levuka	8	5	3	16	5.33	2.05	16	12	7	35	11.67	3.68
Lockes	8	5	2	15	5.00	2.45	15	13	14	42	14.00	0.82
Lona	9	3	5	17	5.67	2.49	16	12	6	34	11.33	4.11
Longmorn	8	5	4	17	5.67	1.70	17	13	9	39	13.00	3.27
Taras	8	5	5	18	6.00	1.41	17	15	9	41	13.67	3.40
Thalia	7	5	5	17	5.67	0.94	17	17	7	41	13.67	4.71
Timo	9	4	4	17	5.67	2.36	16	13	11	40	13.33	2.05
Trevor	9	5	4	18	6.00	2.16	13	13	4	30	10.00	4.24
**Sum**	373	204	189	766			723	593	383	1699		
**Mean**	8.48	4.64	4.50				16.43	13.48	9.12			
**SD**	0.62	0.64	1.55				1.56	1.44	3.03			
